# Expression Patterns of E-Cadherin and N-Cadherin Proteins in the Periodontal Pocket Epithelium of Chronic Periodontitis

**DOI:** 10.30476/dentjods.2022.92474.1652

**Published:** 2023-03

**Authors:** Hamideh Kadeh, Fereshte Arbabi Kalati, Mahdi Ramezaninejad

**Affiliations:** 1 Oral and Dental Disease Research Center, Dept. of Oral & Maxillofacial Pathology, School of Dentistry, Zahedan University of Medical Sciences, Zahedan, Iran; 2 Oral and Dental Disease Research Center, Dept. of Periodontology, School of Dentistry, Zahedan University of Medical Sciences, Zahedan, Iran; 3 School of Dentistry, Zahedan University of Medical Sciences, Zahedan, Iran

**Keywords:** E-cadherin, N-cadherin, Periodontitis, Etiology

## Abstract

**Statement of the Problem:**

E-cadherin and N-cadherin are two types of cell adhesion molecules that are involved in organ development, wound healing, and pathological conditions through the process of epithelial-mesenchymal transition (EMT). However, their role has not yet been fully elucidated in the pathogenesis of periodontal diseases.

**Purpose:**

To determine the expression level of proteins associated with the EMT process (E-cadherin and N-cadherin) in chronic periodontitis.

**Materials and Method:**

In this cross-sectional study, 37 samples (19 cases with healthy gingival tissue and 18 cases with severe chronic periodontitis) that referred to the Periodontology Department of Zahedan Dental School, Zahedan, Iran, in 2018 were included. The samples were immunohistochemically stained with E-cadherin and N-cadherin monoclonal antibodies. Afterward, the percentage of stained cells and the staining intensity of the cells were evaluated. Finally, the obtained data were analyzed using by IBM© SPSS© Statistics version 21 using Mann-Whitney statistical test.

**Results:**

In this study, 89.5% of the healthy gingival tissue samples and 61.1% of samples with chronic periodontitis showed E-cadherin expression in more than 50% of cells. This difference between the two groups was not significant (*p*= 0.13); however, the E-cadherin staining intensity of the healthy gingival tissue was strong while that of the samples with chronic periodontitis was moderate (*p*= 0.002). The N-cadherin expression was negative in 68.4% of healthy gingival cases, while 50% of the cases with chronic periodontitis showed a high expression of N-cadherin. This difference was statistically significant (*p*= 0.04). Moreover, the N-cadherin staining intensity also had a significant difference between the two groups (*p*= 0.004).

**Conclusion:**

Based on the results of the present study, the increased expression of N-cadherin and reduction of staining intensity of E-cadherin was found in chronic periodontitis compared to healthy gingival tissues. Therefore, EMT process may be involved in the pathogenesis of severe chronic periodontitis.

## Introduction

Periodontitis is the sixth most prevalent disease worldwide and is related to oral squamous cell carcinoma [ 1 ]. It is an inflammatory and polymicrobial disease which affects the supporting structure of tooth and it is determined by a lack of epithelial attachment and alveolar bone resorption, which can lead to tooth loss if not treated properly [ 1 - 2 ]. According to the World Health Organization (WHO), 35-50% of the world population suffers from periodontitis [ 3 ].

Junctional and sulcular epithelium are the first barriers versus the dental biofilms, which act as mechanical barriers, they provide the local immune response by discharging inflammatory cytokines and enzymes. Lack of epithelial integrity can be associated with bacterial invasion to the lower connective tissue and potentially increase the inflammatory response and subsequent injury [ 4 ].

Epithelial-mesenchymal transition (EMT) is a rapid and often reversible change from epithelial cell phenotype. This process is essential for proper development during embryogenesis and pathological conditions, such as degenerative fibrosis and cancer [ 5 - 6 ].

During this process, cell phenotypes change from epithelial to mesenchymal, a process that is essentially characterized by the loss of cellular junctions, an increase in mesenchymal markers and a decrease in epithelial markers [ 6 ]. According to previous studies, chronic and persistent inflammation caused by microbial infections can induce EMT in different organs [ 7 - 8 ]. 

It is also possible that EMT starts in the epithelial cells of the mouth during chronic inflammation and leads to the loss of epithelial attachment and epithelial barrier function, and thereby the increase of microbial infiltration in the gingiva and peri-radicular tissues. However, the potential and the exact role of EMT in periodontal diseases, as a mechanism that participates in the epithelial barrier function and oral bacterial infiltration, are not exactly determined [ 9 ].

E-cadherin is a glycoprotein belonging to the cadherin family. It plays an important role in epithelial cohesion through the establishment of epithelial cellular junctions and its absence increases the epithelial cells motility and their ability of local invasion; it is known as an EMT marker in tumor progression. [ 10 - 11 ]. 

N-cadherin (CDH2) is a calcium-dependent adhesion glycoprotein from the cadherin family. This protein is located on the 18q11 chromosome and normally occurs in neuroectodermal and mesodermal tissues. It plays an essential role in the establishment of cellular adhesions in mesenchymal cells. Many studies have indicated the re-expression, increased expression, or decreased N-cadherin expression in human neoplasms and tumor cell categories, especially breast, prostate and thyroid cancer [ 11 - 15 ]. The epithelial cells do not express N-cadherin; however, they could obtain it through cadherin switching process, a process that occurs during EMT and increases cell mobility and invasive properties [ 10 ]. The term of cadherin switching commonly refers to the switching from E-cadherin expression to N-cadherin expression [ 16 ].

Only few researches have been performed on the role of EMT in the pathogenesis of periodontal diseases. Abdulkareem *et al*. [ 2 ] found that long-term exposure of rat oral keratinocytes cultures to periodontal pathogens created EMT-like features; this process might have a role in epithelial integrity loss during periodontitis. However, the role of this process in the pathogenesis of periodontal diseases is not exactly determined [ 2 ]. Therefore, the current study aimed to determine the role of EMT in severe chronic periodontitis through the E-cadherin and N-cadherin proteins expression.

## Materials and Method

### Study design

The Ethics Committee of Zahedan University of Medical Sciences approved the current study (IR.ZAUMS. REC.1397.459). This cross sectional study was conducted on tissue specimens of 37 patients that referred to the Periodontology Department of Zahedan Dental School, Zahedan, Iran, in 2018. The patients who had no history of previous periodontal therapy, systemic or chronic disease, and drug consumption during the past few months were included. Exclusion criteria were defined as smokers, pregnant women, and patients with soft tissue abnormalities. Informed written consents were obtained from the enrolled patients.

### Study groups

The periodontitis group (18 cases) included patients with severe chronic periodontitis who had the signs of loss of attachments >5mm and chronic inflammation changes in periodontium such as bleeding on provocation, redness and change in marginal contour, periodontal pocket formation, and bone resorption. The healthy gingiva tissues (19 cases) were obtained from patients who had undergone crown lengthening surgery, did not have bleeding during mild probing, showed no clinical signs of inflammation, and had a probe depth of 3 mm or less.

### Immunohistochemistry (IHC) and scoring

The samples were cut in the pathology Department and after that hematoxylineosin slides were prepared. The appropriate samples, which had a sufficient amount of tissue, were selected for immunohistochemical (IHC) staining. Immunohistochemistry was performed using the peroxidase Envision polymer detection system (Leica Biosystems, Newcastle, United Kingdom). 

At first, paraffin embedded samples were cut into 4-µm sections, the samples were deparaffinized with xylene and rehydrated with 100%, 90%, and 80% alcohols solution. Antigen retrieval was performed by microwave heating for 30 min on Tris buffer (PH=7.6). Then, the sections were incubated for 1 h at room temperature with primary mouse monoclonal anti-human antibody N- Cadherin Clone 6044777 (Novocastra, United kingdom) at 1:50 dilution (60460-11, Novocastra) and E-Cadherin ready to use antibody Clone 36B5 (Novocastra, United Kingdom) according to the manufacturer’s instructions (Novocastra). After this, the tissue sections were washed at PBS and the secondary antibody was used at room temperature. The diaminobenzidine was added and then counterstained with Mayer’s hematoxylin, dehydrated, and mounted. In negative controls, primary antibody was omitted.

After the IHC staining, the slides were examined using a light microscope (Nikon, Type2, and Tokyo, Japan). The number of stained cells was evaluated at a magnification of 400 in 10 high-power fields (HPF) and categorized as absent, >20%, 20-50%, and >50% for E-cadherin (membranous immunostaining) and as absent, >10%, 10-20%, 20-50% and >50% for N-cadherin (membranous and cytoplasmic immunostaining) [ 17 ]. Intensity of staining was scored as negative (none staining), mild (light brown staining of the cells), severe (dark brown staining of the cells), and moderate (between mild and severe staining of the cells) [ 17 ].

### Statistical Analysis

Data analysis was carried out using IBM© SPSS© Statistics version 21 (IBM© Corp., Armonk, NY, USA). The relationship between the groups was assessed by Mann-Whitney test. *p* Value less than 0.05 were considered statistically significant. 

## Results

**Figure 1:**
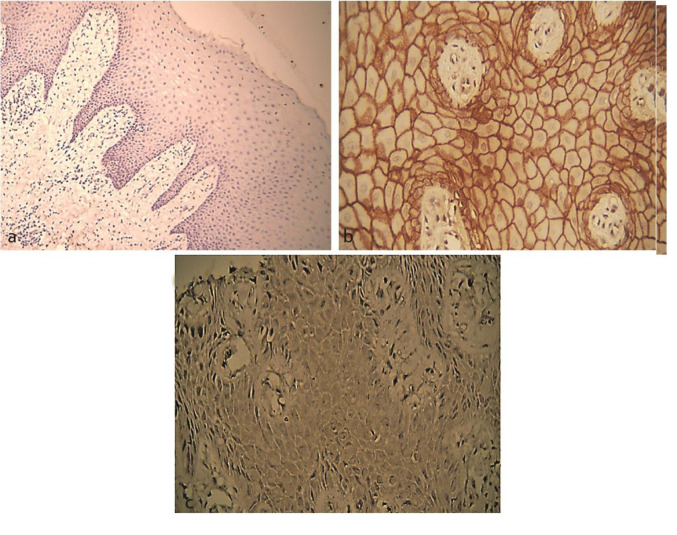
Immunoexpression of E-cadherin and N-cadherin in healthy gingival tissue, **a:** Negative expression of N-cadherin (100×), **b:** Severe expression of E-cadherin (400×), **c:** Mild expression of N-cadherin (400×)

**Figure 2:**
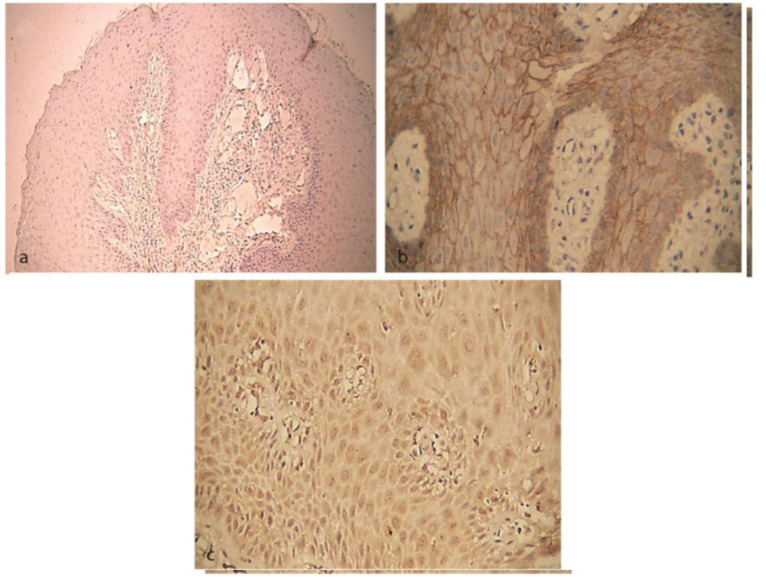
: Immunoexpression of E-cadherin and N-cadherin in chronic periodontitis, **a:** Negative expression of N-cadherin (100×), **b:** Moderate expression of E-cadherin (400×), **c:** Moderate expression of N-cadherin (400×)

The mean ages of the subjects with healthy gingiva and chronic periodontitis were 34.31±5.50 and 41.27± 5.78, respectively. Moreover, 31.6% (n=6) in the groups with healthy gingiva and 27.8% (n=5) of the subjects with chronic periodontitis were male. In terms of the sampling location, 63.2% (n=12) of healthy tissue samples and 61.1% (n=11) of the chronic periodontitis samples were from maxilla. As it is summarized in Table 1, 89.5% (n=17) and 61.1% (n=11) of healthy gingiva and chronic periodontitis samples showed E-cadherin expression in more than 50% of cells. The results of the Mann-Whitney test revealed that the E-cadherin expression in the two groups did not have a statistically significant difference (*p*= 0.13) (Figures 1-2). According to Table 2-4, 68.4% (n=13) of healthy gingiva samples had negative N-cadherin expression and 26.3% (n=5) of the samples showed positive N-cadherin expression in more than 50% of cells. In the group of chronic periodontitis samples, 27.8% (n=5) did not express N-cadherin, while 50% (n=9) of them showed high N-cadherin expression. Based on the results, the expression of N-cadherin in the two groups of healthy and chronic periodontitis samples showed a significant difference (Mann-Whitney Test, *p*= 0.04) (Figures 1-2). Tables 3 and 4 also show the staining intensity of the cells towards E-cadherin and N-cadherin in the studied groups.

**Table 1:** Immune reactivity of E-cadherin in studied groups

Group	E-cadherin expression N (%)					
	Negative	<20%	20-50%	>50%	Total	*p* Value
Healthy gingiva	0(0)	0(0)	2(10.5)	17(89.5)	19(100)	0.13
Chronic periodontitis	0(0)	1(5.6)	6(33.3)	11(61.1)	18(100)	

**Table 2:** Immune reactivity of N-cadherin in studied groups

Group	N-cadherin expression N (%)						
	Negative	<10%	10-20%	20-50%	>50%	Total	*p* Value
Healthy gingiva	13(68.4)	0(0)	0(0)	1(5.3)	5(26.3)	19(100)	0.04
Chronic periodontitis	5(27.8)	0(0)	0(0)	4(22.2)	9(50)	18(100)	

**Table 3:** Staining intensity of E-cadherin in studied groups

Group	Staining Intensity N (%)					*p* Value
	Negative	Mild	Moderate	Severe	Total	
Healthy gingiva	0(0)	0(0)	6(31.6)	13(68.4)	19(100)	0.002
Chronic periodontitis	0(0)	3(16.7)	12(66.7)	3(16.7)	18(100)	

**Table 4:** Staining intensity of N-cadherin in studied groups

Group	Staining Intensity N (%)					*p* Value
	Negative	Mild	Moderate	Severe	Total	
Healthy gingiva	13(68.4)	2(10.5)	4(21.1)	0(0)	19(100)	0.004
Chronic periodontitis	5(27.8)	1(5.6)	6(33.3)	6(33.3)	18(100)	

## Discussion

Maintaining the cohesion of the epithelium performs an essential role in the host defense against pathogens. One of the most prominent criteria in EMT is the reduction of molecules associated with epithelial cell-cell junctions, such as E-cadherin as well as the acquisition and increasing of mesenchymal markers, such as N-cadherin. Consequently, the cells are not polarized and have high mobility [ 10 - 11 ]. This process may be activated by the gram-negative bacteria exposure. It has been suggested that EMT may be in charge for the loss of epithelial barrier function in the pathogenesis of different diseases [ 2 ].

Oral epithelium responds to the periodontal pathogens by secretion of cytokines and chemokines such as tumor necrosis factor (TNF-α), transforming growth factor-β1 (TGF-β1), and epidermal growth factor, which trigger the beginning of EMT [ 7 ]. The effect of EMT in the pathogenesis of periodontitis has not yet been extensively studied. Therefore, the current study investigated the expression of EMT markers (E-cadherin and N-cadherin) in healthy gingiva and chronic periodontitis.

According to the results of the present study, no significant decrease in E-cadherin expression was observed in chronic periodontitis compared to healthy gingival group, but the staining intensity was severe in the majority of the healthy gingival group, while it was moderate in most chronic periodontitis group. In the Nagarakanti *et al*. [ 18 ] study, a significant difference was found between the staining intensity of E-cadherin in the two groups of healthy gingival and periodontitis tissues, which is consistent with this study. Similarly, Ye *et al*. [ 19 ] observed a decrease in the staining intensity of E-cadherin and several other markers in the periodontal pocket epithelium and stated that the ability of the pathological epithelial lining to act as an effective barrier against the entry of bacterial products into the tissue is endangered.

Calcium-based adhesions are among the most common epithelial junctions and are basically made up of smaller units, such as E-cadherin. E-cadherin plays a critical role in the cellular bonds formation; it is complexed with cytokeratin structural proteins and forms a persistent network between epithelial cells resulting in a strong physical barrier [ 2 ]. Therefore, the reduction of E-cadherin expression is the main component of *in vitro* and *in vivo* induction of EMT. Abdulkareem *et al*. [ 2 ] found a reduction in E-cadherin expression in response to stimulation by periodontal pathogens. This process has often been attributed to the gram-negative bacterium *P.gingivalis* [ 2 ]. The results of a study performed by Nagarakanti *et al*. [ 18 ], revealed proteolytic degradation of E-cadherin by *P.gingivalis* and the loss of cellular connections by Hepatocyte Growth Factor (HGF) as the two main causes of the reduction of E-cadherin expression in periodontal diseases. Katz *et al*. [ 20 ] also reported in their *in vitro* study that *P.gingivalis* was able to invade deep connective tissue structures by destroying the epithelial cell-cell junction complex, thereby helping the release of bacteria and destruction of the epithelial barrier through reduction of E-cadherin expression [ 20 ]. In addition, monolayer epithelium permeability in tissue culture increased associated with reduction of E-cadherin expression due to exposure to lipopolysaccharide (LPS) of *P.gingivalis* [ 21 ]. These data are inconsistent with our study that showed no significant decrease in the E-cadherin expression protein in chronic periodontitis tissues, compared to healthy gingival tissues. This is probably due to small sample size and applying sensitive IHC technique in our study. In addition, sample processing and primary antibodies may affect the results.

Microbial pathogens play an essential role in the induction of EMT signaling pathways and mesenchymal phenotype changes. For example, the *Helicobacter Pylori* organism activates EMT through the MAPK/ErK-NF -kB pathways and the suppression of GSK3β activity in gastric cancer. In a study performed by Lee *et al*. [ 22 ], it was found that *P.gingivalis*, as an opportunistic pathogen in prolonged infections in the oral epithelium, was strongly associated with the induction of early changes in EMT. These changes begin with an increase in GSK-3β phosphorylation which, in turn, increases the expression of transcription factors (i.e., Snail, Slug, and Zeb1), followed by a loss and decrease of E-cadherin expression with B-catenin accumulation in oral epithelial cells. These molecular events appear to increase the mesenchymal phenotype and migration in epithelial cells.

In the current study, a considerable increase in N-cadherin expression was found in chronic periodontitis tissues, compared to healthy gingival tissues. There was also a statistically significant difference between the two groups in terms of cell staining intensity towards N-cadherin, which was consistent with the findings of Abdulkareem *et al*. study [ 2 ]. In their study, cultured epithelial cells were exposed to periodontal pathogens (*F. nucleatum* and *P.gingivalis*) and after polymerase chain reaction testing, the E-cadherin expression decreased, while mesenchymal markers expression (N-cadherin, vimentin, Snail1, MMP2) increased [ 2 ]. According to the definition of EMT, one of the main criteria used to define this process is the switching of cadherin from an epithelial phenotype to a mesenchymal phenotype, which is characterized by the simultaneous increase of N-cadherin mesenchymal expression and decrease of epithelial E-cadherin expression. In the present study, this process was observed in 26.7% of cases with chronic periodontitis, which could indicate the role of the EMT process in the pathogenesis of the chronic periodontitis. 

According to the review of the related literature, most previous studies [ 18 - 19 , 21 ] in this field have been limited to the investigation of the effect of periodontal pathogens on E-cadherin expression. Therefore, the expression of epithelial and mesenchymal markers related to EMT (E-cadherin and N-cadherin) has not been adequately investigated. Moreover, there are some limitations in our study, which include the small sample size. Also, the different types of periodontitis, subtypes of microbial colonization and the effect of risk factors associated with periodontitis have not been investigated.

## Conclusion

According to the results of the current study, a decrease in the staining intensity of E-cadherin was observed in the chronic periodontitis tissues compared to the healthy gingival tissues. Moreover, there was a significant increase in the N-cadherin expression in chronic periodontitis group. These results may indicate the possible role of the EMT process in chronic periodontitis pathogenesis. Further investigation on the exact role of periodontal pathogens in inducing EMT and selection of larger samples from different types of periodontitis are needed to explain the clinical significance of this process in periodontitis.

## Acknowledgements

This article was written based on the dataset from a DDS thesis of Mahdi Ramezaninejad with No. 9041 in Zahedan University of Medical Sciences. The authors would like to thank Zahedan University of Medical Sciences for financial support.

## Conflict of Interest

The authors declare that they have no conflict of interest.
